# Prevalence of Frequent Mental Distress and Physical Inactivity Among Adults Aged 18–29 Years — United States, 2012–2024

**DOI:** 10.15585/mmwr.mm7539a1

**Published:** 2026-10-08

**Authors:** Farah Mouhanna, Jasmine Y. Nakayama, Geoffrey P. Whitfield, Jennifer L. Matjasko, Kenneth Rose, Neetu Abad, Morgan Seiler, Courtney Esparza, Craig W. Thomas

**Affiliations:** ^1^Division of Nutrition, Physical Activity, and Obesity, National Center for Chronic Disease Prevention and Health Promotion, CDC; ^2^Epidemic Intelligence Service, CDC; ^3^Office of the Director, National Center for Injury Prevention and Control, CDC; ^4^Children’s Hospital Colorado, Aurora, Colorado; ^5^Healthcare Integration Program Innovation, Portland, Oregon;^ 6^Division of Population Health, National Center for Chronic Disease Prevention and Health Promotion, CDC.

**Summary**

**What is already known about this topic?**

Young adults have the highest incidence of certain mental health conditions. Increasing physical activity has been demonstrated to improve mental health.

**What is added by this report?**

During 2012–2024, the overall reporting of both frequent mental distress and physical inactivity increased nationally among young adults aged 18–29 years; these outcomes either remained unchanged or increased in most jurisdictions. In 2024, 21.3% of young adults reported frequent mental distress and 16.8% reported physical inactivity. Analyses of year-to-year trends identified substantial declines in both outcomes among young adults in 2024.

**What are the implications for public health practice?**

Improving mental health and reducing physical inactivity among young adults through evidence-based strategies could improve public health.

## Abstract

Poor mental health accounts for an estimated $300 billion in medical, pharmaceutical, and workplace- and suicide-related costs in the United States each year; promoting physical activity is an evidence-based approach to improving mental health. Identifying geographic groups with high prevalences of frequent mental distress and physical inactivity could help focus strategies that improve both outcomes. Young adults aged 18–29 years experience the highest prevalence of mental health conditions compared with other adult age groups. Using 2012–2024 data for adults aged 18–29 years from the Behavioral Risk Factor Surveillance System, CDC calculated frequent mental distress and physical inactivity prevalences nationally and by state or territory (jurisdiction). Nationwide, from 2012 to 2024, statistically significant overall increases in the prevalences of frequent mental distress (from 12.7% to 21.3%) and physical inactivity (from 15.9% to 16.8%) were observed. These national changes reflect jurisdiction-level changes from 2012 to 2024, because the prevalence of frequent mental distress increased and physical inactivity prevalence remained unchanged or increased in nearly every jurisdiction. However, examining year-to-year national changes revealed substantial recent declines in both frequent mental distress (during 2022–2023 and 2023–2024) and physical inactivity (during 2023–2024). Improving these outcomes can help prevent chronic diseases, reduce health care costs, and improve overall quality of life. Opportunities exist to improve mental health and increase physical activity concurrently through strategies that include social support groups for physical activity or changes to the physical environment. Such approaches might help sustain recent improvements in mental health and physical activity and lead to overall improvements in health.

## Introduction

Poor mental health is a costly public health concern among U.S. adults. Among various mental health disorders, major depressive disorder alone in the United States accounted for $326.2 billion in medical, pharmaceutical, and workplace- and suicide-related costs in 2018 (expressed in 2020 U.S. dollars) (*1*). In recent decades, among all adults, those aged 18–29 (*2*) and 18–25 years have reported higher prevalences of poor mental health than have those in other age groups. The Physical Activity Guidelines for Americans recognize that physical activity can improve mental health by reducing symptoms of anxiety and depression and improving well-being, cognition, and sleep. Inadequate physical activity is also costly, contributing to an estimated $192 billion (expressed in 2019 U.S. dollars) in adults' annual health care costs, after adjusting for body mass index (*3*).

A strong association has been found between having ever received a diagnosis of a depressive disorder and experiencing frequent mental distress (*4*), defined as ≥14 mentally unhealthy days during the past 30 days, during which the person has experienced poor mental health because of stress, depression, or problems with emotions. CDC’s approach to supporting mental health emphasizes improving conditions where persons work, live, learn, and play. Identifying jurisdictions with increasing prevalences of frequent mental distress and physical inactivity among young adults can help focus evidence-based strategies that can improve both outcomes (e.g., social support and community design for physical activity) in areas of highest need. To identify these areas of highest need, national and jurisdiction-level prevalences of and trends in frequent mental distress and physical inactivity were examined among U.S. adults aged 18–29 years during 2012–2024 using data from the Behavioral Risk Factor Surveillance System (BRFSS).

## Methods

### Data Source and Primary Measures

BRFSS, a state-based landline and cellular telephone survey of noninstitutionalized U.S. civilian residents aged ≥18 years, collects data on health-related risk behaviors, chronic diseases and conditions, health care access, and use of preventive services in all 50 states, the District of Columbia (DC), and participating U.S. territories. Data from 2012 to 2024 were analyzed, limited to years with consistent physical inactivity question placement.* Frequent mental distress was defined as any response of ≥14 days to the question, “Now thinking about your mental health, which includes stress, depression, and problems with emotions, for how many days during the past 30 days was your mental health not good?” Physical inactivity was defined as a negative response to the question, “During the past month, other than your regular job, did you participate in any physical activities or exercises such as running, calisthenics, golf, gardening, or walking for exercise?” 

### Study Participants

The analysis included data from 2012 to 2024 for 424,558 respondents aged 18–29 years. A total of 7,256 respondents with missing answers or who answered “I don’t know” or refused to answer questions on frequent mental distress, physical inactivity, or age were excluded^†^ (Supplementary Table 1).

### Data Analysis

National prevalences of frequent mental distress and physical inactivity were estimated for each study year, as were year-to-year and overall (2012 to 2024) changes. For jurisdiction-level analyses (50 U.S. states, DC, Guam, and Puerto Rico),^§^ 2024 prevalence estimates and change in prevalence from 2012 to 2024 for both outcomes were calculated. Change estimates were considered statistically significant if the 95% CI of the percentage point difference excluded zero.^¶^ All analyses were conducted in R (version 4.4.0; R Studio) using survey weights to account for the complex sampling design and nonresponse.** This activity was reviewed by CDC, deemed not research, and conducted consistent with applicable federal law and CDC policy.^††^

## Results

### National Trends in Frequent Mental Distress and Physical Inactivity (2012–2024)

**Frequent mental distress.** During 2014–2022, the prevalence of frequent mental distress increased statistically significantly in each study year (from 12.4% to 23.7%) (Figure 1). This increase was followed by a 1.2 percentage point (95% CI = 0.2–2.2) statistically significant decrease to 22.5% in 2023 and another 1.2 percentage point (95% CI = 0.3–2.2) statistically significant decrease to 21.3% in 2024. The 2024 national prevalence of frequent mental distress among adults aged 18–29 years corresponds to an overall 8.6 percentage point (95% CI = 7.7–9.4) statistically significant increase from 12.7% in 2012.

**FIGURE 1:**
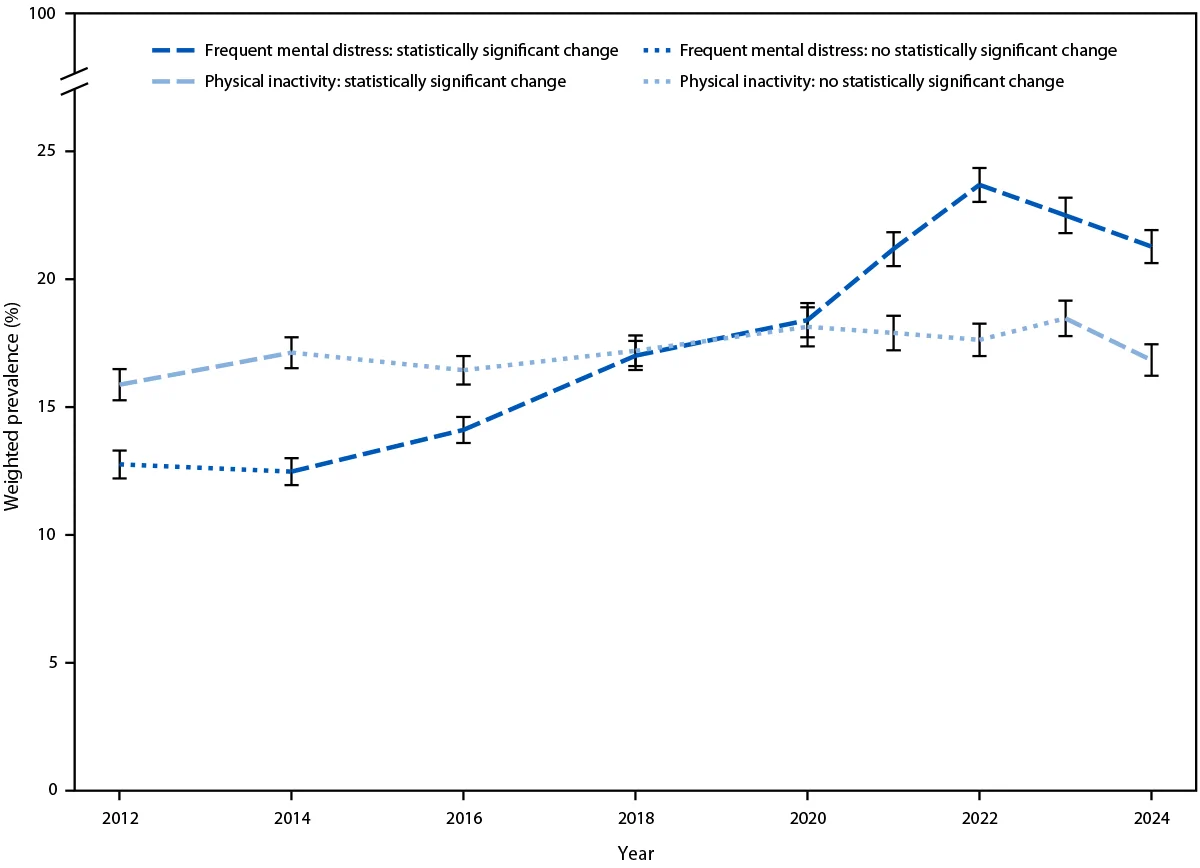
Prevalence* of frequent mental distress^†^ and physical inactivity^§^ among adults aged 18–29 years — Behavioral Risk Factor Surveillance System, United States,^¶^ 2012–2024 * With 95% CIs indicated by error bars. ^†^ Any response of ≥14 days to the question, “Now thinking about your mental health, which includes stress, depression, and problems with emotions, for how many days during the past 30 days was your mental health not good?” ^§^ A negative response to the question, “During the past month, other than your regular job, did you participate in any physical activities or exercises such as running, calisthenics, golf, gardening, or walking for exercise?” ^¶^ National estimates are obtained from respondents from the 50 U.S. states, the District of Columbia, Guam, and Puerto Rico with available Behavioral Risk Factor Surveillance System data.

**Physical inactivity.** During the first 2 years of the analysis period, the prevalence of physical inactivity increased statistically significantly, by 1.3 percentage points (95% CI = 0.4–2.1), from 15.9% in 2012 to 17.1% in 2014. Subsequent year-to-year differences were not statistically significant until the last year of the analysis period, when prevalence declined by 1.6 percentage points (95% CI = 0.7–2.6), from 18.4% in 2023 to 16.8% in 2024. The 2024 national prevalence of physical inactivity (16.8%) corresponds to a 1.0 overall percentage point (95% CI = 0.1–1.8) statistically significant increase from 2012.

### Jurisdiction-Level Prevalences of Frequent Mental Distress and Physical Inactivity (2024)

In 2024, the jurisdiction-level prevalences of frequent mental distress among young adults were highest in Oregon (30.2%), Nevada (27.9%), and Louisiana (27.1%) and lowest in Puerto Rico (6.1%), Guam (14.7%), and Hawaii (14.8%) (Figure 2) (Supplementary Table 2). Physical inactivity prevalences were highest in Puerto Rico (33.8%), Guam (28.5%), and Mississippi (22.1%) and lowest in DC (11.3%), Nevada (11.3%), Montana (11.4%), and Colorado (11.8%).

**FIGURE 2:**
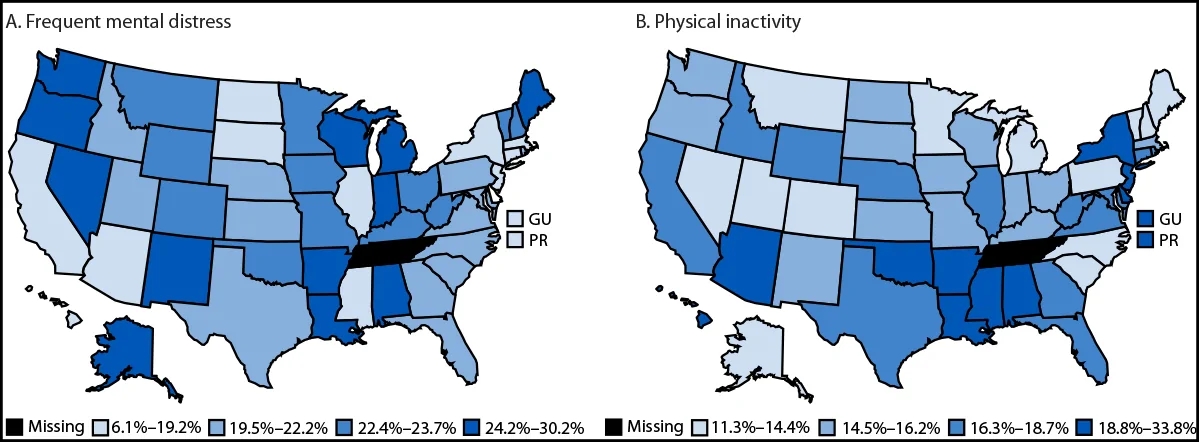
Weighted prevalences of frequent mental distress* (A) and physical inactivity^†^ (B) among adults aged 18–29 years, by jurisdiction — Behavioral Risk Factor Surveillance System, United States, 2024^§^ **Abbreviations: **GU = Guam; PR = Puerto Rico. * Any response of ≥14 days to the question, “Now thinking about your mental health, which includes stress, depression, and problems with emotions, for how many days during the past 30 days was your mental health not good?” ^†^ A negative response to the question, “During the past month, other than your regular job, did you participate in any physical activities or exercises such as running, calisthenics, golf, gardening, or walking for exercise?” ^§^ In 2024, Tennessee did not collect enough data to meet the minimum requirements for inclusion in the public-use dataset.

### Jurisdiction-Level Changes in Prevalences of Frequent Mental Distress and Physical Inactivity (2012–2024)

During 2012–2024, among the 53 jurisdictions included in the analysis, 47 (88.7%) experienced an overall statistically significant increase in prevalence of frequent mental distress (Figure 3) (Supplementary Table 2). Physical inactivity prevalence increased statistically significantly in six (11.3%) of the 53 jurisdictions over the observed time frame; although prevalence declined significantly in Texas, the other jurisdictions had no significant change.

**FIGURE 3:**
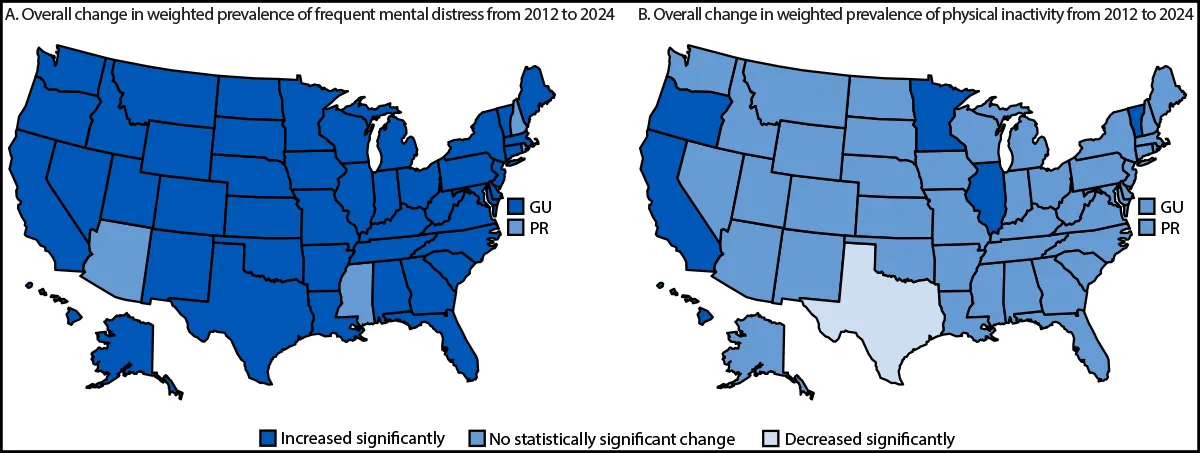
Change in weighted prevalences of frequent mental distress* (A) and physical inactivity^†^ (B) among adults aged 18–29 years, by jurisdiction — Behavioral Risk Factor Surveillance System, United States, 2012–2024^§^ **Abbreviations: **GU = Guam; PR = Puerto Rico. * Any response of ≥14 days to the question, “Now thinking about your mental health, which includes stress, depression, and problems with emotions, for how many days during the past 30 days was your mental health not good?” ^†^ A negative response to the question, “During the past month, other than your regular job, did you participate in any physical activities or exercises such as running, calisthenics, golf, gardening, or walking for exercise?” ^§^ Because 2024 data for Tennessee were unavailable, prevalence estimates were compared from 2012 to 2023.

## Discussion

In 2024, more than one in five (21.3%) young adults reported frequent mental distress, and one in six (16.8%) reported getting no physical activity outside of work in the past month, representing overall increases in both outcomes since 2012. These trends were generally reflected in jurisdiction-level analyses, where in most jurisdictions outcomes were either unchanged or increased from 2012 to 2024. However, year-to-year national trends revealed decreases in more recent years in prevalences of both frequent mental distress (during 2022–2023 and 2023–2024) and physical inactivity (during 2023–2024).

The recent improvements in frequent mental distress align with other recent national trends. For example, prevalences of other indicators of mental health (e.g., major depressive disorder) among persons aged 18–25 years also declined between 2021 and 2024. Both frequent mental distress and physical inactivity could have been affected by the COVID-19 pandemic in the early 2020s through increased loneliness and health anxiety (*5*) and reduced opportunities for physical activity (*6*). Recent declines in frequent mental distress and physical inactivity might reflect reductions in these contributing factors as the pandemic waned.

Improving mental health and lowering the prevalence of physical inactivity can help prevent chronic diseases, reduce health care costs, and improve overall quality of life. One report suggests that from 2010 to 2018, direct medical, pharmaceutical, and workplace- and suicide-related costs for major depressive disorders (which are strongly associated with frequent mental distress) (*4*) among adults aged 18–25 years increased by 73.7% (from $16.4 million to $28.5 million, expressed in 2020 U.S. dollars) (*1*). Not meeting the aerobic physical activity guideline (which includes physical inactivity) is associated with $192 billion (expressed in 2019 U.S. dollars) in annual health care expenses among U.S. adults (*3*). Reductions in mental health symptoms or disorders (e.g., depression and anxiety) (*7*) and participation in regular physical activity are associated with improved quality of life.

Opportunities exist to concurrently improve mental health and physical activity to sustain recent improvements. Although the relationship between these two outcomes can be bidirectional (e.g., poor mental health could be a barrier to physical activity) (*8*), the Physical Activity Guidelines for Americans recognize that physical activity improves cognition and reduces the risk for depression and anxiety. Thus, concurrent improvements in both mental health and physical activity to sustain recent improvements are possible. For example, referrals to spend more time in nature by a health or social professional can promote physical activity, and time in nature also promotes mental well-being, even in the absence of physical activity (*9*). Walking groups and buddy systems can increase physical activity and forge social connectedness. In addition, community design interventions, including increasing neighborhood walkability and access to green spaces, have been linked to increases in physical activity and improved mental health (*10*).

### Limitations

The findings in this report are subject to at least five limitations. First, the self-reported outcomes were subject to reporting and social desirability biases, possibly resulting in underestimates of frequent mental distress and physical inactivity. Second, single-item survey questions measuring the multifaceted constructs of mental health and physical activity do not capture more complex details (e.g., type and severity of mental health conditions, duration of physical activity, or job-related physical activity). Third, jurisdictional estimates should be considered exploratory, because the CIs for many jurisdictions were wide, and multiple comparisons increased the likelihood of chance findings. Fourth, the analysis did not explore whether a respondent reported both frequent mental distress and physical inactivity, which limited analysis and interpretation to the independent prevalence estimates of these distinct but often interrelated health outcomes. Finally, the cross-sectional design prevents inferences regarding causality, especially considering the recognized bidirectional association between mental health and physical activity (*8*).

### Implications for Public Health Practice

Despite recent declines in the prevalences of frequent mental distress and physical inactivity among U.S. adults aged 18–29 years in recent years, a substantial percentage of young adults report frequent mental distress and physical inactivity. Implementing strategies that can improve both physical activity and mental health, including several in CDC’s Active People, Healthy Nation Initiative, might reinforce these recent declines. Relevant examples include promoting increased time in nature, using social support groups to increase physical activity, and designing safe and convenient access to supportive community environments for physical activity.

## Footnotes

* Years of data included in the analysis are 2012 (response rate = 49.1% for the landline telephone–administered survey and 35.3% for the cellular telephone–administered survey), 2014 (response rate = 48.7% [landline] and 40.5% [cellular]), 2016 (response rate = 47.7% [landline] and 46.4% [cellular]), 2018 (response rate = 53.3% [landline] and 43.4% [cellular]), 2020 (combined landline and cellular weighted response rate = 47.9%), 2021 (combined response rate = 44.0%), 2022 (combined response rate = 45.0%), 2023 (combined response rate = 44.7%), and 2024 (combined response rate = 43.9%). BRFSS Annual Survey Data | CDC

† The demographic characteristics (sex and race or ethnicity) of included and excluded respondents were calculated across all years. Most differences between included and excluded respondents were small except for the percentage of Hispanic or Latino (Hispanic) and non-Hispanic White (White) populations (17.8% of included and 21.9% of excluded [Hispanic], and 59.6% of included and 51.1% of excluded [White] respondents). Sex and race or ethnicity were not variables in the main study, because the study's aims were to describe prevalence over time and geography.

§ Tennessee did not collect sufficient data for BRFSS 2024 to meet the minimum requirements for inclusion in the public-use dataset. For Tennessee, 2023 prevalence estimates and change in prevalence during 2012–2023 are reported instead of 2024 prevalence estimates and change in prevalence during 2012–2024.

¶ SE of the change estimate was calculated as the square root of the sum of the squared SEs from the 2 years compared. The 95% CI for the change estimate was calculated as the change estimate plus or minus 1.96 times SE of the change estimate.

** A report describing the weighting methodology is available on the BRFSS website for each data year (e.g., for year 2024: Complex Sampling and Weights and Preparing 2024 BRFSS Module Data for Analysis).

†† 45 C.F.R. part 46.102(l)(2), 21 C.F.R. part 56; 42 U.S.C. Sect. 241(d); 5 U.S.C. Sect. 552a; 44 U.S.C. Sect. 3501 et seq.
